# Plant Root-Exudates Recruit Hyperparasitic Bacteria of Phytonematodes by Altered Cuticle Aging: Implications for Biological Control Strategies

**DOI:** 10.3389/fpls.2020.00763

**Published:** 2020-06-09

**Authors:** Sharad Mohan, K. Kiran Kumar, Vivek Sutar, Supradip Saha, Janet Rowe, Keith G. Davies

**Affiliations:** ^1^Division of Nematology, Indian Council of Agricultural Research, Indian Agricultural Research Institute, New Delhi, India; ^2^Indian Council of Agricultural Research, Central Citrus Research Institute, Nagpur, India; ^3^Division of Agricultural Chemicals, Indian Council of Agricultural Research, Indian Agricultural Research Institute, New Delhi, India; ^4^Plant Pathology and Microbiology, Rothamsted Research, Harpenden, United Kingdom; ^5^Department of Biological and Environmental Sciences, University of Hertfordshire, Hatfield, United Kingdom; ^6^Norwegian Institute of Bioeconomy Research, Ås, Norway

**Keywords:** co-evolution, host-parasite interaction, Red Queen dynamics, *Pasteuria*, endospore adhesion, *Meloidogyne*, *Heterodera*

## Abstract

Phytonematodes are globally important functional components of the belowground ecology in both natural and agricultural soils; they are a diverse group of which some species are economically important pests, and environmentally benign control strategies are being sought to control them. Using eco-evolutionary theory, we test the hypothesis that root-exudates of host plants will increase the ability of a hyperparasitic bacteria, *Pasteuria penetrans* and other closely related bacteria, to infect their homologous pest nematodes, whereas non-host root exudates will not. Plant root-exudates from good hosts, poor hosts and non-hosts were characterized by gas chromatography-mass spectrometry (GC/MS) and we explore their interaction on the attachment of the hyperparasitic bacterial endospores to homologous and heterologous pest nematode cuticles. Although GC/MS did not identify any individual compounds as responsible for changes in cuticle susceptibility to endospore adhesion, standardized spore binding assays showed that *Pasteuria* endospore adhesion decreased with nematode age, and that infective juveniles pre-treated with homologous host root-exudates reduced the aging process and increased attachment of endospores to the nematode cuticle, whereas non-host root-exudates did not. We develop a working model in which plant root exudates manipulate the nematode cuticle aging process, and thereby, through increased bacterial endospore attachment, increase bacterial infection of pest nematodes. This we suggest would lead to a reduction of plant-parasitic nematode burden on the roots and increases plant fitness. Therefore, by the judicious manipulation of environmental factors produced by the plant root and by careful crop rotation this knowledge can help in the development of environmentally benign control strategies.

## Introduction

Belowground biodiversity plays a key role in determining ecological and evolutionary outcomes to terrestrial ecosystems, and nematodes, which are globally abundant, play a key function in determining local patterns of soil fertility (Bargett and Van Der Putten, 2014; Van Den Hoogen et al., 2019). Plant-parasitic nematodes are an important component of the root herbivore community where multitrophic interactions between their host plant and their natural enemies determine plant productivity in both natural and economically important agricultural systems (Van Der Putten et al., 2006). Soil bacteria are another abundant and diverse group of soil microorganism important in ecosystem processes that remain largely functionally and taxonomically uncharacterized because the majority are uncultivatable in the laboratory (Lok, 2015; Delgado-Baquerizo et al., 2018). *Pasteuria penetrans* (ex. Thorne) Sayre and Starr and other closely related species of bacteria are obligate hyperparasites of phytonematodes and have been shown to be successful in the belowground suppression of plant-parasitic nematodes (Davies, 2009). Aboveground studies of hyperparasites shows them to be important in regulating crop pathogens and pests (Bianchi et al., 2006; Parratt and Laine, 2018) and understanding the mechanisms of their recruitment by host plants has led to novel systems of crop protection (Cook et al., 2007). Although it has been argued that aboveground and belowground interactions can range from the mutually beneficial to the mutually detrimental (Jagodič et al., 2019), currently we lack sufficient examples to understand the eco-evolutionary perspectives of the below ground ecology of the multitrophic rhizosphere (Wardle et al., 2004). Here we explore a belowground tri-trophic interaction between plant hosts, their nematode parasites using homologous and heterologous bacterial endospore forming hyperparasites, *Pasteuria* spp. with the view to develop new crop protection strategies for phytonematodes.

Recent comparisons of transcriptomes between the plant-parasitic nematodes *Globodera rostochiensis* (Behrens, 1975) Behrens, 1975 and *Globodera pallida* (Behrens, 1975) Behrens, 1975 treated with tomato and potato root diffusates have revealed species differences in gene expression during the initiation of hatch (Duceppe et al., 2016). The nematode cuticle, a protective barrier against microbes, is a secreted product of the hypodermis; it is subject to aging (Herndon et al., 2002), and studies show it has an inducible defense response against bacterial pathogens (Darby et al., 2002; Ewbank, 2002; Gravato-Nobre and Hodgkin, 2005). The Gram-positive endospore forming bacterium of the *Pasteuria* group is a potential biological control agent for plant-parasitic nematodes and the initiation of its infection process is the binding of endospores to the surface coat of the cuticle of infective juveniles (Davies, 2009). The host parasitic interaction between *Daphnia* spp. and the Gram-positive endospore forming bacterial micro-parasite *Pasteuria ramosa* Metchnikoff has become a valuable model for studying co-evolutionary relationships where it is reported that the binding of endospores, a key stage in the infection process, is genetic and under negative frequency-dependent selection (also known as “Red Queen dynamics”) and not linked to environmental factors (Decaestecker et al., 2007; Ebert et al., 2016). However, meta-barcoding studies in Scottish soils suggest that *Pasteuria* communities are structured and correlate with environmental factors including soil carbon, moisture and pH (Orr et al., 2020) suggesting and environmental component to this nematode hyperparasite interaction.

It is known that *Pasteuria* endospores that encumber nematodes bind differentially to populations of infective juvenile nematodes and there is most certainly a genetic component to this (Davies et al., 1994; Davies, 2009), however, it is also well known that whilst the cuticle is known to be heterogeneous it is also highly labile (Davies et al., 2001; Davies and Curtis, 2011). Given that root-diffusates have long been known to alter nematode hatching and behavior (Ellenby and Perry, 1976), we hypothesized that the surface coat with its heterogeneous nature and lability may be under inducible control from environmental factors such as root exudate. Using co-evolutionary theory and the aboveground recruitment of hyperparasites by plants (Bianchi et al., 2006; Cook et al., 2007; Parratt and Laine, 2018), we therefore conjecture that the binding of hyperparasitic endospores is likely to be increased by host root exudates of nematode susceptible host plants compared to exudates from non-host plants; and that environmental biotic factors, in the form of root-diffusate, may have a functional role in the recruitment of endospores in this tri-trophic interaction to the plants benefit.

## Materials and Methods

### Nematodes

Pure cultures of *Meloidogyne incognita* (Chitwood, 1949) Chitwood, 1949 and *Heterodera cajani* Koshi, 1967 were maintained on tomato (*Solanum lycopersicum* cv. Pusa Ruby) and cowpea [*Vigna unguiculata* (L.) Walp. cv. Pusa Komal] plants grown in sterilized soil and sand in the ratio of 1:1; maintained in a glasshouse at 26°C (±4) with 12 h day/night diurnal period. The egg masses of individual nematodes were collected from the infected plants and kept for hatching in sterile water at 28°C. The second-stage infective juveniles (J2) which hatched within 24 h were considered fresh (T_0_), while remaining were left in water for 7 days (T_7_) and 14 days (T_14_) to age.

### Pasteuria

The J2 of the two nematodes were encumbered with their specific *Pasteuria* isolates (MiPp and HcPn) using the centrifugation method (Hewlett and Dickson, 1993), and inoculated near the root zone of tomato and cowpea seedlings. After 35 days, the roots were gently washed and the infected *M. incognita* females were retrieved by dissecting the galls and infected females were picked out using a pair of forceps. Individual females were crushed and observed under a compound microscope to confirm the presence of endospores which were transferred to 1.5 mL eppendorf tube and a stock of 5 × 10^6^ endospores per mL water prepared and stored in refrigerator at 4°C for further use.

### Extraction of Exudates and Gas Chromatography-Mass Spectrometry (GC/MS) Analysis

A procedure developed by adapting those of Aulakh et al. (2001) and Valentinuzzi et al. (2015) to collect exudates. Seeds of cowpea cv. Pusa Komal, tomato cv. Pusa Ruby and potato cv. Kufri Chandramukhi were sterilized with 70% ethanol for 30 s, followed with 1% sodium hypochlorite for 1 min, and finally washed three times with distilled water; then sown in sterilized 3:1 soil and sand mixture. Emerged seedlings were allowed to grow for 4 weeks in a glass house at 26°C (±4)/12 h day/night rhythm. Plants were watered with sterilized water every second day till they were uprooted and washed gently in sterile deionized water. The roots of intact plants were submerged in 500 mL glass beakers containing 400 mL deionized water of HPLC quality for 10 h exudation at 25°C. Exudates from 5 beakers, each containing 5 plants were pooled to make 2 liters of final sample which was stored at 4°C and used for endospore encumbrance assays and GC-MS analysis for the profiling of the root exudates after partitioning with hexane. GC-MS analysis was carried out using 7890A GC (Agilent Technologies, United States) equipped with a HP-5MS column (30 m × 0.25 mm; 0.25 μm, Agilent Co., United States) connected to a triple axis HED-EM 5975C mass spectrometer (Agilent Co., United States). The injection volume was 1 μL with flow mode in split control, while the carrier gas flow was set at 1 mL min^–1^ helium. Helium (High Purity, New Delhi, India) was used as carrier gas at a head pressure of 10 psi. For the analysis, oven temperature was initially held at 40°C for 1 min, thereafter, raised with a gradient of 3°C min^–1^ until it reached 60°C, and held for 10 min. The temperature was again raised with a gradient of 2°C min^–1^ up to 220°C and held for 1 min. Finally temperature was raised up to 280°C with an increment of 5°C min^–1^ with total runtime of 111 min. The MS acquisition parameters were as follows: ion source 180°C, electron ionization 70 eV, full scan mode (50–550 mass units), transfer line temperature 280°C, solvent delay 3 min, and EM voltage 1376. The ionization energy was 70 eV with a scan time of 1 s and mass range of 50–550 AMU. Compounds were identified by matching their mass spectra. NIST (National Institute of Standards and Technologies) Mass Spectra Library was used as a reference for identifying each component.

### Attachment Assays

Treatment of the J2s of the two species of species of plant-parasitic nematodes with root exudate was undertaken by placing them in a cavity block containing 1 mL of undiluted root exudate and they were incubated at 28°C for 24 h. Standardized endospore attachment bioassays of the homologous hyperparasites (HcPn and MiPp) were performed with their host nematodes (PpCN and RKN), respectively, in siliconized microcentrifuge tubes by mixing 50 μL stock suspensions of endospores (500 μL^–1^) with 50 μL of nematodes (4 μL^–1^) and centrifuging the organisms together at 8000 rpm for 5 min (Hewlett and Dickson, 1993). Endospores adhering to each species of the J2 were measured using a high-powered bright-field microscope (×400). Two replicates were performed in two microcentrifuge tubes containing approximately 200 J2 and endospore attachment was assessed by counting the spores adhering to 20 nematodes from each tube; each of the two replicate microcentrifuge tubes attachment bioassays were performed at least twice in time. Endospore attachment assay on cuticle aging was performed as described above on T_0_, T_7_, and T_14_ infective juveniles in distilled water on both nematode species *H. cajani* and *M. incognita*.

### Scanning Electron Micrograph

The attachment of *Pasteuria* endospores to the nematodes was performed as described above by centrifugation for scanning electron micrograph (SEM). Briefly, spore encumbered nematodes were placed on Whatman^TM^ filter paper attached to a cryo stub with OCT mountant (Sakura Finetek, Europe, Netherlands), frozen using liquid nitrogen and transferred under vacuum to the microscope for observation (Mohan et al., 2012). Scanning was undertaken using a JEOL (United Kingdom) JSM 6700 FEH scanning electron microscope at −160°C and images captured with JEOL on board image system and software.

## Results

### Scanning Electron Microscopy and Host Range

**FIGURE 1:**
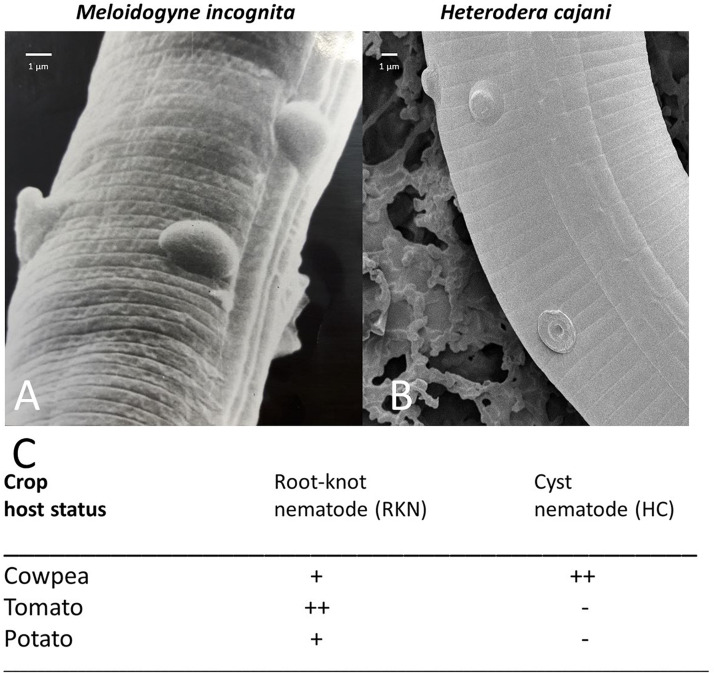
**(A)** Scanning electron micrograph (SEM) of an infective juvenile of the root-knot nematode *Meloidogyne incognita* (RKN) encumbered with endospores of *Pasteuria penetrans*; **(B)** SEM of an infective juvenile of the cyst nematode *Heterodera cajani* (HC) encumbered with endospores of a *Posterior nishizawae*-like species; **(C)** the crop status as a major host (++ economically important and affects crop yield), minor host (+ not economically important with little effect on crop yield), none host (– does not infect plant), to the plant nematodes *H. cajani* and *M. incognita* as designated from the CABI species datasheets (www.cabi.org/isc/datasheet/27023 and www.cabi.org/isc/datasheet/33245, respectively).

Our experimental system uses two species of plant-parasitic nematodes (Figures 1A–C). *M. incognita* (Figure 1A) a parthenogenetic species of root-knot nematode (RKN) which is polyphagous and can infect a wide range of host crops, and *H. cajani* (Figure 1B) pigeon pea cyst nematode (PPCN) which reproduces amphimictically and is specific to pigeon-pea, cowpea, and other closely related legumes; each of these plant-parasitic nematodes is host to the *Pasteuria* hyperparasite; *P. penetrans* (Davies et al., 2001) and *Posterior nishizawae* Sayre, Wergin, Scmidtt, and Starr (Mohan et al., 2012), respectively. Endospores of *Pasteuria* isolated from *H. cajani* did not adhere to juveniles or infect *M. incognita*, and endospores from *M. incognita* do not adhere to or infect *H. cajani*.

### Effect of Cuticle Age on Endospore Attachment

**FIGURE 2:**
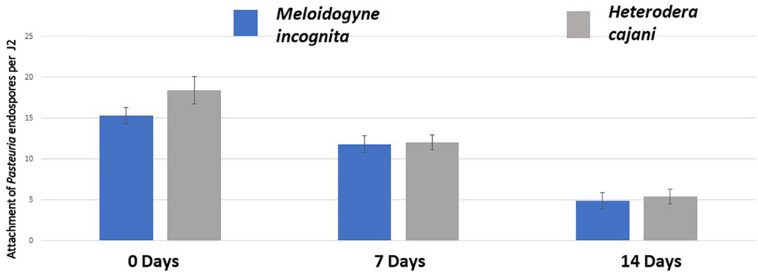
The mean number of endospores adhering to the cuticle of infective juveniles of Meloidogyne incognita (RKN) and Heterodera cajani (He) at 0, 7, and 14 days following a standardized attachment bioassay (ANOVA *P* = 1.15^−12^; bar is ± standard error of the mean; each treatment *n* = 20 × 2 replicates undertaken twice in time).

There was no statistical significant difference between RKN and PPCN in the number of endospores adhering to the cuticles of either RKN and PPCN at any one time point (Figure 2); however, over time there was a reduction from over 15 spores J2^–1^ at T_0_ to approximately 12 spores J2^–1^ at T_7_ and 5 spores J2^–1^ at T_14_; this reduction was statistically significant (ANOVA *P* < 0.001) and shows that as the cuticles of both species of infective juveniles mature over time they become less susceptible to endospore adhesion.

### Effect of Different Plant Root Exudates on Endospore Attachment

**FIGURE 3:**
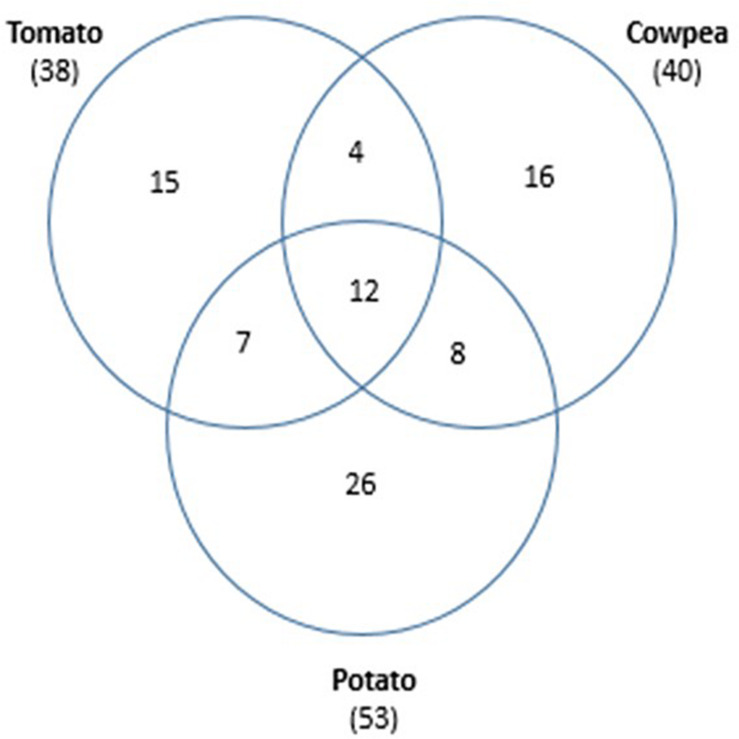
Venn diagram of the 88 compounds identified by GC/MS contained in root exudates in common from the solanaceous crops, tomato, and potato and the Legume, cowpea; numbers in parentheses are the total number of compounds identified in the crop plant (table of compounds identified see Supplementary Material).

To explore the effect of plant root exudates on the nematode cuticle and identify the compounds that may be responsible for the recruitment of hyperparasitic *Pasteuria* through increased endospore adhesion, root exudates from the legume cowpea (*V. unguiculata*, cv. Pusa Komal) and the solanaceous crops, tomato (*S. lycopersicum*, cv. Pusa Ruby), and potato (*Solanum tuberosum*, cv. Kufri Chandramukhi) were collected and GC/MS analysis revealed a large number of compounds (Figure 3 and Supplementary Table 1) many of which it was impractical to investigate in detail as standards were not available. In total 88 individual compounds were putatively identified of which 15 and 16 individual compounds were specific to tomato and cowpea, respectively. A further 26 individual compounds were associated solely with potato. Twelve compounds were common to all three crops, with seven compounds shared between the solanaceous crops, tomato, and potato; eight more were shared between potato and cowpea. And only four compounds were in common between cowpea and tomato (Figure 3).

### Effects of Different Root Exudates on the Attachment of *Pasteuria* Endospores

**FIGURE 4:**
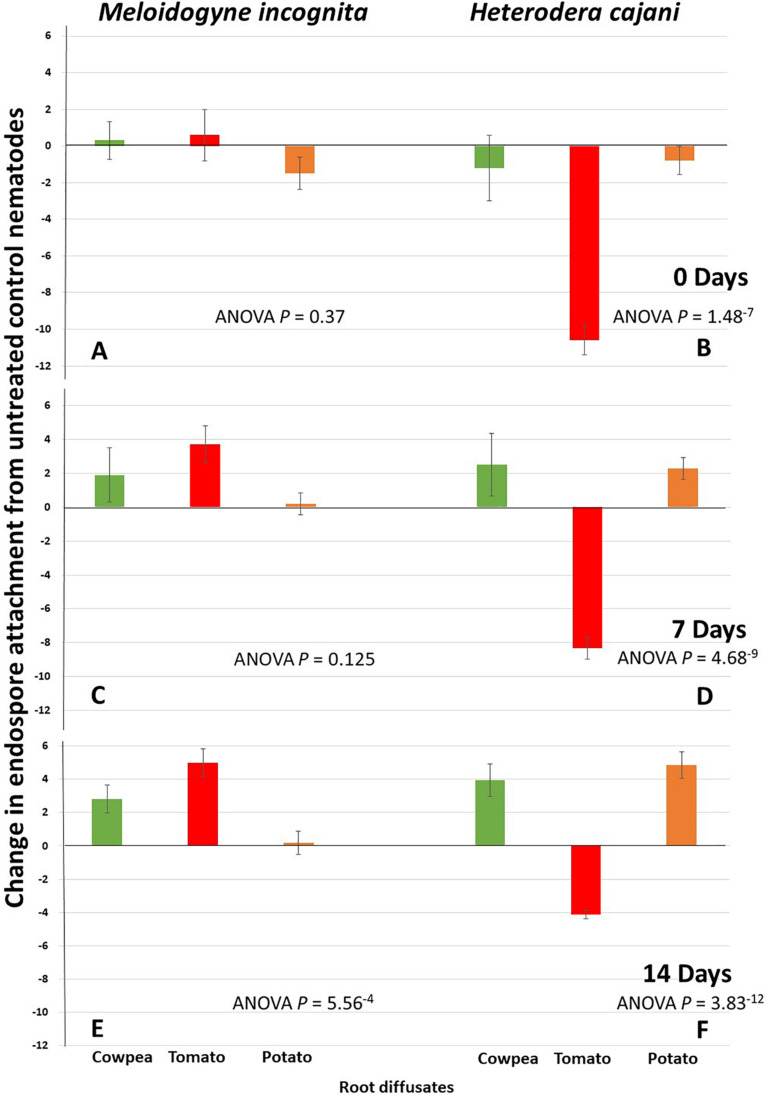
Mean change of endospore attachment of tPasteuria (spores J2^–1^) to infective juveniles of Meloidogyne incognita and Heterodera cajani from a water treated controls at 0 **(A,B)**, 7 **(C,D)**, and 14 **(E,F)** days post hatch following treatment with root diffusates from different host plants of cowpea, tomato, and potato (error bars are standard error of the mean; each treatment *n* = 20 × 2 replicates undertaken twice in time).

There was a gradual increase in the statistical significance of RKN treated with cowpea and tomato root exudates over time (Figure 4). Although there was a general reduction in endospore attachment in both nematode species over time (Figure 2), root exudates from the hosts for *M. incognita*, cowpea and tomato, led to changes in endospore attachment from less than one at T_0_ (Figure 4; ANOVA *P* = 0.37), to between one and four, respectively, at T_7_ (ANOVA *P* = 0.125); this increased to over two and four endospores per juvenile, respectively, at T_14_ (ANOVA *P* = 5.56^–4^) days post hatch (Figure 4) showing that as the cuticle matured the root exudates resulted in an increased adhesion of hyperparasitic endospores. However, the effects of potato root diffusate, which was negative at T_0_ and near zero at T_7_ and T_14_ was clearly only marginal (Figure 4). Tomato root exudate, obtained from plants that grow well in both tropical and sub-tropical climates and is highly susceptible to *M. incognita*, appears to have had the greatest effect in enhancing endospore attachment of its homologous *Pasteuria* population and root exudates from cowpea, a poor host for *M. incognita*, still enhanced endospore adhesion but to approximately half the extent at T_7_ and T_14_, respectively (Figure 4; ANOVA *P* = 0.125 and *P* = 5.56^–4^).

The exposure of *H. cajani* juvenile nematodes to root diffusates on endospore attachment was striking; the effect of tomato root diffusate, a non-host for *H. cajani*, resulted in a large reduction in endospore attachment ranging from reductions of around 10 spores per nematode compared to control at T_0_, to reductions of 8 at T_7_, and 4 at T_14_ similarly. These compared very differently to the effects of root diffusate from cowpea, the host plant, and potato, a non-host plant where both these root diffusates had a marked increase in endospore attachment ranging from approximate reductions of one spore per nematode compared to controls at T_0_, but increases of over 2 spores at T_7_, and 4 spores or above at T_14_ (Figure 4; ANOVA *P* ≤ 1.48^–7^).

## Discussion

We show that plant root exudates from a host plant changed the susceptibility of the nematode cuticle to endospore adhesion by affecting the natural cuticle maturation process; these changes led to hyperparasitic recruitment in the homologous system using host root exudates, whereas in the heterologous system, using non-host root exudates, this was not the case. Using our tri-trophic model we contrasted the effects of host root-diffusates from roots of plants of the polyphagous *Meloidogyne* against root diffusates from the more plant host specific *H. cajani* and the subsequent effects on endospore attachment to nematode cuticle. The co-evolutionary model developed using the *Pasteuria – Daphnia* spp. system invoked “Red Queen dynamics” (Decaestecker et al., 2007; Ebert et al., 2016), and we might therefore expect a similar model to play a key role in *Pasteuria –* nematode interactions. However, the host-plant – phytonematode – *Pasteuria* model is likely to be more complex due to its tri-trophic nature, and such multi-trophic interactions would perhaps be expected to model the hyperparasitic interactions of above ground pests of plants where hyperparasites can be recruited by plant signals (Bianchi et al., 2006; Parratt and Laine, 2018). Moreover, if correct, we would therefore expect that root exudates from a host plant to increase endospore adhesion to the cuticle of infective juvenile nematodes of a plant-parasitic nematode, whereas non-host root exudates would not. The phenomenon of cuticle maturity of RKNs being linked to a reduction of endospore attachment is not new (Davies et al., 1991); however, this phenomenon has not been investigated in such detail before. The model system described here enables us to explore the tritrophic interactions between plant-host root diffusates, the cuticle aging process of two very different plant-parasitic nematode genera (*Meloidogyne* and *Heterodera*, both economically globally important), and the hyperparasitic bacterium *P. penetrans* and a close relative. Although, potato can be a host to *M. incognita* it is generally grown in more temperate climates and in terms of plant-nematode co-evolution, other species of nematode would have had the competitive edge, e.g., the amphimictic root-knot and cyst nematodes which prefer cooler conditions, and which therefore would be unlikely to be exposed to any long-term selection pressure in nature.

Experiments with *H. cajani* a cyst nematode with a restricted host range reveals some interesting differences when compared to the polyphagous *M. incognita*. What is immediately striking is that root exudates from tomato, a non-host plant, have a huge detrimental effect of endospore attachment at all time points (Figure 4); this is most marked at T_0_, but as the cuticle ages and although the differences become smaller they become increasingly statistically significant. The host-root exudates from cowpea increase endospore attachment in line with the recruitment hypothesis at T_7_ and T_14_, but interestingly, so did the root exudate from potato, a non-host and therefore not expected to recruit endospores. This runs counter to our hypothesis and is noteworthy; what is exciting is that the polyphagous *M. incognita* reproduce parthenogenetically and although comparisons of endospore attachment between sexual and asexual populations of *Meloidogyne* spp. have revealed that there are mechanisms to maintain variability in asexually reproducing populations (Davies et al., 2008), it is likely that the surface cuticle of sexually reproducing populations, such as *H. cajani*, are likely to have a larger degree of variability, and it is indeed especially interesting that the population of *Pasteuria* from *H. cajani* also adheres to and infects potato cyst nematodes (Mohan et al., 2012), suggesting some form of inter- and intra-specific genetic mechanisms that generate surface coat variation that is different between root-knot and cyst nematodes. However, both are manipulatable through root exudates of the host plant by affecting the cuticle maturation process in favor of endospore recruitment.

Individual compounds putatively identified by GC/MS showed differences in the exudates from the different plant hosts (Figure 3 and Supplementary Table 1) but identifying the ones responsible for manipulating the cuticle aging process proved inconclusive. This perhaps was not surprising as it was impractical to even attempt to identify some of the compounds as standards were not available. Of those given provisional identifications, 12 compounds were common in all three root exudates and can likely be eliminated as responsible for changing cuticle maturation. It might have been expected that the compounds identified in the solanaceous crops would be similar when compared to the leguminous crop, but in fact tomato and cowpea exudates were broadly similar with 38 and 40 compounds, respectively. Potato had a total of 53 compounds of which 26 were individual to potato, that is approximately 10 more than either tomato or cowpea. With respect to *M. incognita*, cuticle aging as measured by endospore attachment, responded to root exudates of both tomato and cowpea which shared four compounds (Supplementary Table 1: 1-Hexadecene; Nonadecene; octadecanoic acid and 3,5-di-tert-Butyl-4-hydroxyphenylpropionic acid) any, or a combination of several different ones, may have a regulatory effect on the cuticle aging process. *H. cajani* cuticle responded to both cowpea and potato root exudate which had eight compounds in common (Supplementary Table 1: Eicocene; 3-Eicocene; Cyclohexadecane; Docosane; E-15-Heptadecenal; 1,2-Benzenedicarboxylic acid, bis(2-methylpropyl) ester; Dibutyl phthalate and 4-Nonyl-phenol). Another important consideration is that although methods were adopted to maintain these exudates in sterile conditions, this proved difficult and microbial contamination cannot be excluded; therefore, some of these compounds may be extracellular compounds of microbial origin. This does not necessarily negate these results as crops are grown in soil from which they select their own rhizosphere microbiome (Hirsch and Mauchline, 2012; Lundberg et al., 2012; Rolfe et al., 2019) and these compounds may also have a key role in nematode cuticle maturation. It is well recognized that plants produce a huge number of secondary metabolites and the roots are no exception. Clearly the role of root exudates is an area that needs more attention; firstly, perhaps by GC/MS using an increased array of known standards, but also using other approaches, for example, exploiting model plants with mutations known to affect secondary metabolite production.

A series of diverse collagen-like proteins, postulated to have been acquired by horizontal gene transfer, have been identified in genome of *P. penetrans* and are thought responsible for endospore attachment (Srivastava et al., 2019). To date, no cuticle receptor for collagen-like protein binding has been identified, but experiments with *Caenorhabditis elegans* have identified several mutants (designated *srf, bus*, and *bah*) which are involved in building the complex glycoconjugates of the nematode surface coat and which affect microbial pathogenicity (Gravato-Nobre and Hodgkin, 2005, 2011); one such mutation, *bus-4*, confers bacterial resistance by the production of altering mucins (Parsons et al., 2014). Interestingly, knockdown of a RKN mucin gene using RNAi with infective juveniles of *M. incognita* was also found to reduce *P. penetrans* endospore attachment (Phani et al., 2018) suggesting its involvement, but similar RNAi knockdown experiments targeting another gene (Mi-FAR-1) also affected endospore adhesion to the cuticle (Phani et al., 2017). This suggest that adhesion of endospores is the result of a complex set of cuticular interactions, and it is highly likely that the molecules responsible for the variation exhibited by endospore attachment to the cuticle may also have a function in the nematode – plant interaction; for example, ascaricides, compounds originating in the nematode cuticle and responsible for trap formation in nematode hyperparasitic fungi, also have a role in the nematode – plant interactions (Manosalva et al., 2015; Manohar et al., 2020). Moreover, our observation that root-exudates differentially affect cuticle aging, together with the fact that RNAi knockdown experiments can modulate endospore attachment strongly suggests the belowground multitrophic interactions of the rhizosphere are amenable to experimental manipulation.

**FIGURE 5:**
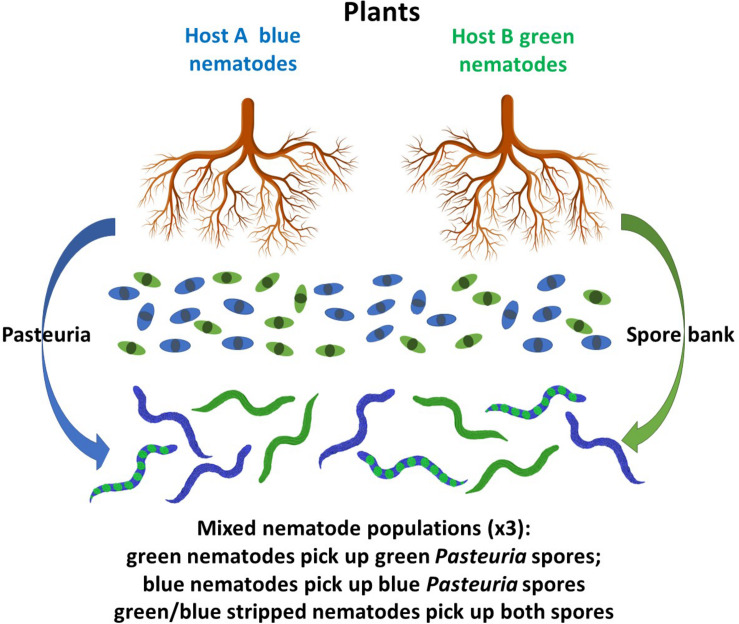
Cartoon representing a theoretical model of the interactions between plant root exudates (arrows) and the cuticle of nematodes: blue arrow represents exudates that recruit *Pasteuria* endospores to the blue nematodes by a reduction in cuticular aging, they also decruit endospore adhesion to the green nematodes by increased cuticular aging; similarly, the green arrow represents exudates that decrease cuticula r aging to the green nematodes thereby recruiting green endospore attachment and increase cuticular aging in the blue nematodes and decruit endospore adhesion; exudates that decruit endospore adhesion maintain those spores in the *Pasteuria* spore bank to control nematodes to which both spores types can attach and can infect both host plants represented by the blue/green stripped nematodes producing an arms in the effects of root exudates (not to scale).

Based on our results we have developed a model (Figure 5) that in summary form shows that environmental factors, such as root exudates from a phytonematode host plant, can recruit the hyperparasitic bacterium by reducing the rate at which the cuticle ages in comparison to non-host root exudates which did not; additionally, we have shown that root exudates from non-host plants have an increased maturation effect on the cuticles of phytonematodes that are not homologous parasites of those plants by decruiting the hyperparasitic endospores. The effect of this is to thereby maintain them in the *Pasteuria* spore bank where they remain available to bind to other phytonematode hosts. Using these results, we have proposed a hypothetical working model of endospore attachment through the modulation of cuticle maturation (Figure 5). Co-evolutionary theory invoking *Red Queen* dynamics (negative frequency-dependent selection) would suggest that the overall population dynamics of each organism in the interaction is genetic and a product of the various bilateral arms races going on between the various components of this multi-trophic interaction, however, there is clearly an environmental component as reflected by the observation that root exudates affect cuticle aging. The overall molecular nature of these interactions and their environmental regulation remains elusive, but insights can be gained from mechanistic genetic approaches using RNAi.

The role of root exudates, including the possible function of their containing phytohormones, has been shown to affect *Pasteuria* endospore attachment (Liu et al., 2017) and changes in host specificity between *Pasteuria* and the phytonematode community occur on a yearly basis (Liu et al., 2019); necessarily, this is the result of population regulatory outcomes from bottom-up control through the host plant, horizontal control of competition between phytonematodes, and topdown control by natural enemies (Van Der Putten et al., 2006). The importance of root-exudates in a plant’s adaptive defense mechanism by maintaining a protective microbiome is an active area of investigation (Rolfe et al., 2019) and here, from a perspective of eco-evolutionary dynamics, we propose a model in which the role of the plant root exudates on the cuticular aging process is fundamental as they will be locked into a co-evolutionary arms race involving rhizosphere signaling directly between the host plant and its nematode parasite and indirectly by differentially affecting hyperparasitic recruitment. Similarly, the heterogeneous and labile nature of the surface coat of the infective juvenile cuticle, which is sloughed off during migration of the nematode in the plant root (Davies and Curtis, 2011), is also likely to act as an effector and trigger plant immune responses (Topalovic et al., 2020). The evolution of these multitrophic interactions and the mechanisms involved have been the result of natural ecosystem evolution that has remained undisturbed over long periods of time, and this contrasts sharply with agriculture which artificially determines the crop and cultivar on a seasonal basis. Recent successful aboveground crop protection systems have exploited multitrophic interactions to develop novel *push-pull* pest control strategies (Cook et al., 2007); whether such systems can be employed for belowground control of plant-parasitic nematodes remains a challenge. Our working model presented here suggests that phytonematode cuticular age is influenced by root exudates and is a key determinant in the co-evolutionary outcomes of the multitrophic population dynamics of rhizosphere ecology.

## Data Availability Statement

The data that support the findings of this study are available from the corresponding author upon reasonable request.

## Author Contributions

SM and KD conceived and designed the experiments and wrote the initial draft of the manuscript. All authors contributed to the interpretation of the data and manuscript preparation. SM, KK, and VS undertook management of the nematode, bacterial cultures, plants, and performed the attachment experiments. SS undertook the GC/MS analysis of the root-exudates. JR was responsible for the electron microscopy and has now retired from Rothamsted Research.

## Conflict of Interest

The authors declare that the research was conducted in the absence of any commercial or financial relationships that could be construed as a potential conflict of interest.
