# Supplementary material for: Plant Root-Exudates Recruit Hyperparasitic Bacteria of Phytonematodes by Altered Cuticle Aging: Implications for Biological Control Strategies
Source: Front Plant Sci. 2020 Jun 9;11:763. doi: 10.3389/fpls.2020.00763 (PMC7296116; doi:10.3389/fpls.2020.00763)
Supplement: Supplementary file 1 [file Table_1.DOCX]

**Supplementary Table 1: Components identified from plant root exudates by GC/MS**

| **Name of compound** | **Molecular formula** | **Molecular weight (g/mol)** | **Tomato** | **Potato** | **Cowpea** |
| --- | --- | --- | --- | --- | --- |
| **Hydrocarbons** | | | | | |
| Dodecane | C_12_H_26_ | 170.34 | **✓** | **✓** | **✓** |
| 1-Tetradecene | C_14_H_28_ | 196.37 | **✓** | **✓** | **✓** |
| Tetradecane | C_14_H_30_ | 198.39 | **✓** | **✓** | **✓** |
| Pentadecane | C_15_H_32_ | 212.42 | **✓** | **✓** | **✓** |
| 1-Hexadecene | C_16_H_32_ | 224.42 | **✓** | - | **✓** |
| Hexadecane | C_16_H_34_ | 226.45 | **✓** | **✓** | **✓** |
| Heptadecane | C_17_H_36_ | 240.48 | **✓** | **✓** | **✓** |
| Cyclopentadecane | C_15_H_30_ | 210.39 | - | - | **✓** |
| Hexadecane, 2,6,10,14-tetramethyl- | C_20_H_42_ | 282.54 | - | - | **✓** |
| 1-Octadecene | C_18_H_36_ | 252.49 | **✓** | **✓** | **✓** |
| Octadecane | C_18_H_38_ | 254.49 | - | - | **✓** |
| Eicosane | C_20_H_42_ | 282.54 | - | **✓** | **✓** |
| Nonadecane | C_19_H_40_ | 268.52 | - | - | **✓** |
| 3-Eicosene, (E)- | C_20_H_40_ | 280.53 | - | **✓** | **✓** |
| Cyclohexadecane | C_16_H_32_ | 224.42 | - | **✓** | **✓** |
| 1-Docosene | C_22_H_44_ | 308.58 | **✓** | **✓** | **✓** |
| Docosane | C_22_H_46_ | 310.6 | - | **✓** | **✓** |
| Heneicosane | C_21_H_44_ | 296.57 | - | - | **✓** |
| Nonadecene | C_19_H_38_ | 266.50 | **✓** | - | **✓** |
| 1-Hexacosene | C_26_H_52_ | 364.69 | - | - | **✓** |
| Cyclotriacontane | C_30_H_60_ | 420.79 | **✓** | **✓** | **✓** |
| Undecane | C_11_H_24_ | 156.31 | - | **✓** | - |
| Nonane | C_9_H_20_ | 128.2 | - | **✓** | - |
| Tridecane | C_13_H_28_ | 184.3614 | **✓** | **✓** | - |
| Heneicosane | C_21_H_44_ | 296.574 | - | **✓** | - |
| Tricosane | C_23_H_48_ | 324.62 | **✓** | **✓** | - |
| 1-Hexadecene | C_16_H_32_ | 224.42 | - | **✓** | - |
| Cyclotetradecane | C_14_H_28_ | 196.38 | - | **✓** | - |
| Tetratriacontane | C_34_H_70_ | 478.91 | - | **✓** | - |
| Cyclopentadecane | C_15_H_30_ | 210.39 | - | **✓** | - |
| 5-Octadecene | C_18_H_36_ | 252.49 | - | **✓** | - |
| Hexacosane | C_26_H_54_ | 226.45 | - | **✓** | - |
| Cyclooctacosane | C_28_H_56_ | 392.744 | **✓** | **✓** | - |
| Dodecane, 2,6,11-trimethyl- | C_15_H_32_ | 212.41 | **✓** | - | - |
| 1-Eicosene | C_20_H_40_ | 280.53 | **✓** | - | - |
| Z-14-Nonacosane | C_29_H_60_ | 408.78 | **✓** | - | - |
| Z-5-Nonadecene | C_19_H_38_ | 266.50 | **✓** | - | - |
| Pentane, 3-methyl- | C_6_H_14_ | 86.18 | - | - | **✓** |
| Pentane, 2,4-dimethyl- | C_7_H_16_ | 100.201 | - | - | **✓** |
| Pentane, 3-methylene- | C_6_H_12_ | 84.15 | - | - | **✓** |
| **Ketones** | | | | | |
| Ar-tumerone | C_15_H_20_O | 216.31 | **✓** | **✓** | **✓** |
| 7,9-Di-tert-butyl-1-oxaspiro(4,5)deca-6,9-diene-2,8-dione | C_17_H_24_O_3_ | 276.37 | **✓** | **✓** | **✓** |
| 7-[Methoxy]-2,2,8-trimethyl-4-chromanone | C_13_H_16_O_3_ | 220.26 | - | - | **✓** |
| 2-Piperidinone | C_5_H_9_NO | 99.13 | - | **✓** | - |
| Cyclopenten-4-one | C_9_H_14_O | 138.20 | - | **✓** | - |
| 2-Cyclohexen-1-one | C_6_H_8_O | 96.127 | **✓** | **✓** | - |
| 2,6-di-butyl-2,5-cyclohexadiene-1,4-dione | C_14_H_20_O_2_ | 220.30 | - | **✓** | - |
| 7-Ethoxy-2,2-dimethyl-4-chromanone | N/A | N/A | **✓** | **✓** | - |
| Loganin aglycone | C_11_H_16_O_5_ | 228.24 | **✓** | - | - |
| 3,5-di-tert-Butyl-4-hydroxyacetophenone | C_16_H_24_O2 | 248.36 | **✓** | - | - |
| 1-Phenyl-3-(L-Threo Triacetoxypropyl)-4,5 Pyrazoledione-4-Phenylhydrazone | N/A | N/A | **✓** | - | - |
| **Aldehydes** | | | | | |
| E-15-Heptadecenal | C_17_H_32_O | 252.43 | - | **✓** | **✓** |
| Nonanal | C_9_H_18_O | 142.24 | - | **✓** | - |
| **Esters** | | | | | |
| i-Propyl tetradecanoate | C_17_H_34_O_2_ | 270.45 | - | - | **✓** |
| 1,2-Benzenedicarboxylic acid, bis(2-methylpropyl) ester | C_16_H_22_O_4_ | 278.34 | - | **✓** | **✓** |
| Dibutyl phthalate | C_16_H_22_O_4_ | 278.34 | - | **✓** | **✓** |
| Isopropyl myristate | C_17_H_34_O_2_ | 270.45 | **✓** | **✓** | - |
| tert-Butyl 8-Methyl-10-azabicyclo[ 4.3.1]deca-3,7-diene-10-carboxylate | C_15_H_23_NO_2_ | 249.34 | - | **✓** | - |
| 3-(4 Bromophenyl)propenoic acid, ethyl ester | C_11_H_11_BrO_2_ | 255.108 | - | **✓** | - |
| Di-n-octyl phthalate | C_24_H_38_O_4_ | 390.56 | - | **✓** | - |
| Methyl 2- pentenoate | C_6_H_10_O_2_ | 114.14 | - | **✓** | - |
| Diisobutyl phthalate | C_16_H_22_O_4_ | 278.34 | **✓** | - | - |
| Diisooctyl maleate | C_20_H_36_O_4_ | 340.49 | **✓** | - | - |
| Dotriacontyl heptafluorobutyrate | C_36_H_65_F_7_O_2_ | 662.88 | **✓** | - | - |
| **Phenols** | | | | | |
| Phenol, 4-nonyl- | C_15_H_24_O | 220.35 | - | **✓** | **✓** |
| Phenol, bis(1,1-dimethylethyl)- | C_14_H_22_O | 206.32 | - | **✓** | - |
| Phenol, 2,4-bis(1,1-dimethylethyl)- | C_14_H_22_O | 206.32 | **✓** | - | - |
| **Amines** | | | | | |
| 13-Docosenamide, (Z)- | C_22_H_43_NO | 337.58 | - | - | **✓** |
| 9-Octadecenamide | C_18_H_35_NO | 281.47 | - | **✓** | - |
| **Fatty acids** | | | | | |
| Hexadecanoic acid | C_16_H_32_O_2_ | 256.42 | **✓** | **✓** | **✓** |
| Octadecanoic acid | C_18_H_36_O_2_ | 284.47 | **✓** | - | **✓** |
| 3,5-di-tert-Butyl-4-hydroxyphenylpropionic acid | C_17_H_26_O_3_ | 278.39 | **✓** | - | **✓** |
| **Alcohols** | | | | | |
| 1-Octadecanethiol | C_18_H_38_S | 286.55 | - | - | **✓** |
| **Heterocyclic organic compounds** | | | | | |
| Dibenzofuran | C_12_H_8_O | 168.19 | - | **✓** | - |
| **Sesquiterpenes** | | | | | |
| .beta.-Bisabolene | ‎C_15_H_24_ | 204.36 | - | **✓** | - |
| **Others** | | | | | |
| Furan, tetrahydro-2-methyl- | C_5_H_10_O | 86.13 | - | - | **✓** |
| Citronellyl Valerate | C_15_H_28_O_2_ | 240.38 | - | **✓** | - |
| HAHNFETT | N/A | N/A | - | **✓** | - |
| Celidoniol | C_29_H_60_ | 408.6 | - | **✓** | - |
| 1H-Pyrazole,4,5-dihydro-5,5-dimethyl-4 isopropylidene- | C_8_ H_14_N_2_ | 138.21 | **✓** | - | - |
| 1-Hentetracontanol | C_41_H_84_O | 593.10 | **✓** | - | - |
| Artonin L monomethyl ether | N/A | N/A | **✓** | - | - |
| Oxirane | C_2_H_4_O | 44.05 | - | **✓** | - |
| 1,2,3-Trimethyldiaziridine | C_4_H_10_N_2_ | 86.135 | - | - | **✓** |
| trans-2,3-Epoxydecane | C_10_H_20_O | 156.26 | - | - | **✓** |
| 1-Bromoheptadecane-1-13C | CH_3_(CH_2_)_16_Br | 319.36 | **✓** | **✓** | **-** |
| 1-Chloroeicosane | C_20_H_41_Cl | 316.99 | - | **✓** | **-** |
| 2-Methyltricos-3-ene | C_24_H_5_O | 338.65 | **✓** | **-** | **-** |
